# Pregnancy With a Bicornuate Uterus Complicated by Placenta Percreta and Intraperitoneal Hemorrhage

**DOI:** 10.7759/cureus.54519

**Published:** 2024-02-20

**Authors:** Reem Talal Almehzaa, Amala Sunder, Nayla Bushaqer

**Affiliations:** 1 Obstetrics and Gynaecology, Bahrain Defence Force Hospital, West Riffa, BHR; 2 Obstetrics and Gynaecology, Royal College of Surgeons in Ireland - Bahrain, Busaiteen, BHR

**Keywords:** müllerian duct, placenta percreta, intraperitoneal hemorrhage, uterine rupture, bicornuate

## Abstract

Uterine malformations significantly affect the reproduction process, and such anomalies can affect the progression and prognosis of a pregnancy. A bicornuate uterus is a rare congenital uterine anomaly that occurs due to a defect in the fusion of Müllerian ducts. It is associated with severe maternal and fetal complications, such as uterine rupture, vascular-related pathologies, preterm labor and birth, recurrent early or late loss of pregnancy, and fetal growth restriction. In such scenarios, close monitoring and ultrasound screening are needed to prevent obstetric complications. We report a case of a bicornuate uterus complicated with placenta percreta and intraperitoneal hemorrhage.

## Introduction

A bicornuate uterus forms as a result of the abnormal fusion of the Müllerian duct, which is the primordial anlage for the uterus, cervix, fallopian tubes, and superior part of the vagina. A bicornuate uterus results in two separate but communicating endometrial cavities, usually connected to the same cervix and vagina. Most women with Müllerian duct anomalies may not face a problem with fertility because they have functioning ovaries. However, because Müllerian duct anomalies are associated with difficulty in maintaining a pregnancy and its evolution, these abnormalities are associated with high-risk complications, including mortality of the mother or baby [1-3]. Moreover, it is crucial to raise patients’ awareness of such conditions and their complications and provide patients with psychological support [4,5].

## Case presentation

The patient was a 36-year-old gravida 7 para 4 with two previous miscarriages. She had a known case of bicornuate uterus unicollis, each attached to one tube and ovary. She was booked in our maternity unit for her current pregnancy from early pregnancy and started following up in our department at eight weeks and one day.

The patient’s past obstetric history included her first two pregnancies, which ended with first-trimester miscarriages, followed by a preterm normal vaginal delivery at 26 weeks that resulted in an early neonatal death. In her next three pregnancies, she had cervical cerclage and a lower-segment cesarean section. Her first cesarean section was performed at 28 weeks for the indication of growth restriction at 760 g and resulted in early neonatal death. The second cesarean section was a preterm breech and labor at 32 weeks, and her most recent delivery was with a cesarean section at 36 weeks of gestation due to a breech in labor. During the current pregnancy, cerclage was done at 13 weeks. Progesterone support was provided, and she was planned for a cesarean section and tubal ligation at 36 weeks because she had a very thin lower segment in her previous surgery.

At 21 weeks, she was brought by ambulance to the emergency maternity unit with unprovoked, painless, severe vaginal bleeding. An ultrasound showed a single active fetus. The fetal heart rate was 152 beats per minute, the liquor was normal, the placenta was anterior in the uterine wall, low and covering the cervix, and the estimated fetal weight was 355 g. The cervix was 2.3 cm in length. On per speculum examination, active vaginal bleeding was noted.

The patient was admitted, kept under observation, and the bleeding was stopped. Admission hemoglobulin was 9 g/dL, and she was hemodynamically stable. On day two of admission, the patient suddenly developed hypovolemic shock and was escalated to the intensive care unit. Her hemoglobin level was 4.6 g/dL despite continuous transfusion, and her abdomen was distended. An ultrasound showed a massive intraperitoneal collection. An alive active fetus was seen with a good amount of liquor, and the uterine contour was intact. She was proceeded for an emergency laparotomy with high-risk consent. The blood bank was alerted for a massive transfusion, and she was resuscitated with blood and blood products such as fresh frozen plasma and cryoprecipitate. A multidisciplinary team, including an obstetrician, surgeons, vascular surgeon, and urologist, were involved in the procedure.

Intraoperative findings

**Figure 1:**
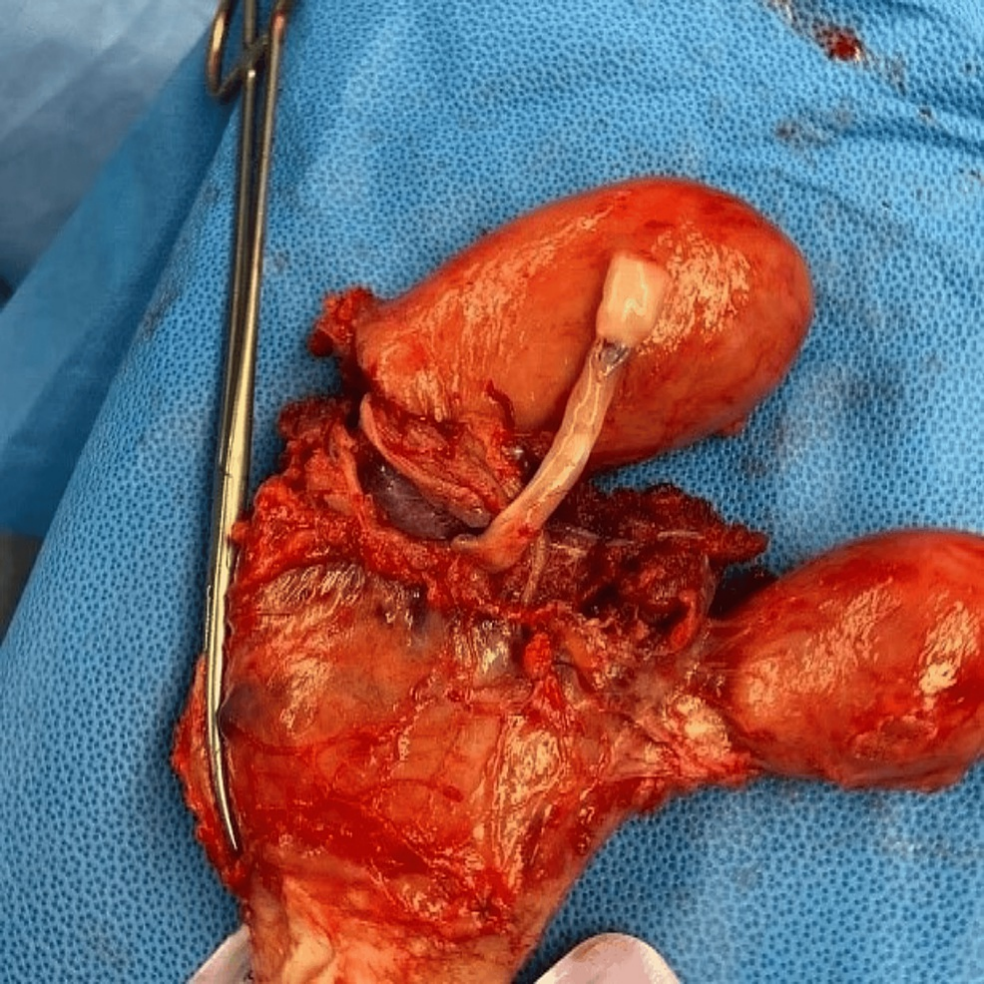
Bicornuate uterus with placenta percreta.

A massive intraperitoneal hemorrhage was noted. The patient has a bicornuate unicollis uterus and placenta percreta invading through the septum between both hemi-uteri, which was bleeding. Uterine bodies and the previous scar were intact. The bladder was intact, and there was no invasion of the placenta to the bladder. A hysterotomy was performed. Upon extraction, the baby was not alive, and the placenta was completely adherent and covering the internal os of the cervix. The operation was converted to a hysterectomy, and both uteri with the septal invasion of the placentae, as shown in Figure 1. Both ovaries were retained.

A bilateral ureteric ligation was confirmed by an endoscope and RG study by the urologist, one of the complications encountered during the procedure. The patient received a massive blood transfusion and blood products intraoperatively. The total estimated blood loss was approximately 4 L. The team secured hemostasis with sutures and cauterization.

A right ureteric implantation was done, and a bilateral double J stenting was done. The left ureteric ligation was released. A cystoscopy confirmed the left ureteric patency. Hemostatic material and fibrillar were kept in the vaginal vault and the raw area, and complete hemostasis was secured.

Postoperatively, she was kept in the intensive care unit for two days and extubated on the second day. After two days, she was transferred to the regular postoperative ward and had a good recovery. She was on redivac for one week and a Foley catheter for six weeks in view of the ureteric injury. She was admitted for 20 days and was discharged home in stable condition, clinically improved, with a hemoglobin of 10.7 g/dL. The patient was discharged with a Foley catheter, which was removed six weeks postoperatively. Double J stent and renal implantations were removed two months later, and the patient is currently doing well with no active complaints.

## Discussion

Failure or disturbances during the fusing phase of the Müllerian ducts leads to the development of uterine abnormalities, and one of these abnormalities is a bicornuate uterus. This rare malformation can lead to maternal morbidities due to acute emergencies, as seen in our patient. However, rupture of scar pregnancy could be considered a differential diagnosis. Other complications include menstrual irregularities, infertility, and abnormal uterine bleeding [6].

Diagnosis can be before, after, or during pregnancy, and, in some cases, the condition may remain unidentified because the patient can be asymptomatic. The condition may become an incidental finding intraoperatively or after developing related obstetric complications such as uterine rupture or complications during labor leading to a cesarean section. The complications have a risk of hysterectomy [6], as seen in our case.

In this case, the patient was a known case of bicornuate uterus, and she had already suffered the complications of this anomaly in her previous pregnancies. This pregnancy was complicated by placenta previa and an intraperitoneal hemorrhage. Despite complications with this type of malformation, successful pregnancies are possible [7].

In cases where the patient is a known case of bicornate uterus, surgical treatment can be advised before pregnancy. For example, the patient may undergo a reunification for a bicornuate uterus known as Strassman metroplasty, or a removal of the septum known as Tompkins or Jones metroplasty for a septate uterus. Such procedures can improve the patient’s prognosis and outcome in future pregnancies [8,9].

In cases where such anomalies are detected during pregnancy, close surveillance is the only management option. In some cases where cervical length is short, which is common in patients with bicornuate uterus, cervical cerclage is advised to maintain the pregnancy and decrease the risk of a late miscarriage or preterm birth [10].

## Conclusions

Uterine anomalies and malformations are difficult to assess or predict, and the exact incidence can never be accurate because patients can be asymptomatic, with outcomes and complications varying from case to case. Complications of such anomalies can range from preterm delivery to uterine ruptures and massive blood loss and mortality. Having a prenatal diagnosis of this kind of malformation can provide better care by being proactive during the pregnancy. Keeping in mind differential diagnoses such as ruptured scar pregnancy can improve the outcome for both the mother and child. In the current practice, women with such conditions can have a normal, uneventful pregnancy, but it is important to be aware of and anticipate the complications that may arise with this condition. Wise thinking and tailored management by the obstetrician can aid successful outcomes.
